# Implementing Ultrasound-Guided Microwave Ablation for Benign Thyroid Nodules in ENT Practice: Technical Considerations and Early Clinical Experience

**DOI:** 10.3390/jcm15187053

**Published:** 2026-09-11

**Authors:** Konstantinos Chaidas, Maria Zisoglou, Chrysovalantis Stylianou, Dimitrios Askitis, Hippokrates Moschouris, Michael Katotomichelakis

**Affiliations:** 1 Ear, Nose, and Throat (ENT) Department, University Hospital of Alexandroupolis, Medical School, Democritus University of Thrace, 68100 Alexandroupolis, Greece; maria.ziso@hotmail.com (M.Z.); mkatotom@med.duth.gr (M.K.); 2 Radiology Department, University Hospital of Alexandroupolis, 68100 Alexandroupolis, Greece; chrys.stylianou@gmail.com (C.S.); hipmosch@gmail.com (H.M.); 3 Private Practice for Endocrinology, 68100 Alexandroupolis, Greece; dimitrios.askitis@gmail.com

**Keywords:** microwave ablation, thyroid nodule, thermal ablation, ultrasound-guided ablation, benign thyroid nodule, otorhinolaryngology, minimally invasive treatment

## Abstract

**Background/Objectives:** Ultrasound-guided microwave ablation (MWA) has emerged as an effective minimally invasive treatment for benign thyroid nodules, but practical guidance regarding implementation of the technique within otorhinolaryngology practice remains limited. This study aimed to describe the implementation of ultrasound-guided MWA for benign thyroid nodules in a tertiary ENT center, with emphasis on technical considerations, procedural safety, and early clinical outcomes. **Methods:** A retrospective analysis of prospectively collected data included consecutive patients seen at the ENT outpatient clinic between March 2024 and July 2025. All procedures were performed by a single ENT surgeon, and technical aspects were described. Procedural characteristics, pain, complications, nodule volume reduction, compressive symptoms, cosmetic outcomes, and thyroid function were evaluated through 3-month follow-up. **Results:** Twenty-five patients underwent ultrasound-guided MWA for 30 benign thyroid nodules. All procedures were successfully completed under local anesthesia. No major complications occurred; two patients developed minor, self-limiting ecchymosis. Median nodule volume decreased from 12.77 mL (IQR, 4.13–17.74) at baseline to 3.81 mL (IQR, 1.31–7.60) at 3 months, corresponding to a mean volume reduction ratio of 63.8%. Significant improvements in compressive symptoms and cosmetic scores were observed (*p* < 0.001). All patients remained euthyroid, with no cases of new-onset hypothyroidism. **Conclusions:** This early experience supports the feasibility and short-term procedural safety of ultrasound-guided MWA for selected benign thyroid nodules within an ENT setting. Careful patient selection, meticulous technique, and structured operator training appear to be key components for successful implementation. Beyond its favorable early clinical outcomes, this study provides a practical framework for centers seeking to establish thyroid MWA programs.

## 1. Introduction

The growing application of high-resolution ultrasonography (US) has significantly increased the detection rates of thyroid nodules, with prevalence ranging from 19% to 68% in the general population [1,2]. Although the vast majority of thyroid nodules are benign, accounting for approximately 80–95% of cases, a substantial number of patients require therapeutic intervention, when nodules become symptomatic or cosmetically evident [3].

Clinical indications for treatment include compressive symptoms such as dysphagia, dyspnea, globus sensation, cosmetic deformity, and psychological distress associated with fear of malignant transformation. For decades, surgery remained the standard treatment option for symptomatic benign thyroid nodules. However, the potential risk of complications, including recurrent laryngeal nerve injury, hypocalcemia, postoperative hypothyroidism, skin scarring, and prolonged recovery, has stimulated interest in minimally invasive alternatives [4,5].

Thermal ablation techniques, including radiofrequency ablation (RFA), laser ablation (LA), high-intensity focused ultrasound (HIFU), and microwave ablation (MWA), have increasingly gained acceptance as organ-preserving therapeutic options [6,7]. Among these techniques, MWA has emerged as a promising modality, because of its intrinsic physical advantages related to microwave energy. Clinical studies have consistently demonstrated favorable efficacy and safety following MWA for benign thyroid nodules [8,9,10], leading to incorporation of MWA into international guidance [11]. Despite the growing international experience with thyroid thermal ablation, published data regarding the implementation of MWA in ear, nose, and throat (ENT) practice remain limited. Furthermore, standardization of technical aspects and procedural safety strategies remains an area of ongoing interest.

The aim of the present study is to describe the implementation of US-guided MWA for benign thyroid nodules in a tertiary ENT center, with emphasis on patient selection, technical considerations, and early clinical experience. To the best of our knowledge, this study also represents the first published Greek clinical series of thyroid MWA.

## 2. Materials and Methods

### 2.1. Study Design

This study represents a retrospective analysis of prospectively collected data from consecutive patients with benign thyroid nodules evaluated at the outpatient ENT clinic of the University Hospital of Alexandroupolis in Greece, between March 2024 and July 2025. The data cutoff for the present analysis was November 2025, by which time all included patients had completed at least 3 months of follow-up. Although follow-up was scheduled at 6 and 12 months, these time points were not uniformly available across the entire cohort at the data cutoff. Accordingly, the present analysis reports technical implementation, procedural safety, and early clinical outcomes through 3 months. All procedures were performed by the same ENT surgeon (K.C.), thereby ensuring procedural consistency throughout the study. The study protocol was approved by the Institutional Review Board of University Hospital of Alexandroupolis (ID:16553) and informed consent was obtained from all participants.

#### 2.1.1. Patient Eligibility

Patients were considered eligible if they fulfilled the following criteria: 1. Age >18 years old; 2. Benign ultrasonographic appearance (EU-TIRADS 2–3); 3. Benign cytology (Bethesda II) confirmed by at least one fine-needle aspiration biopsy (FNAB) or two in the presence of US features warranting additional confirmation before ablation in accordance with current recommendations [12]; 4. Presence of a single or dominant thyroid nodule, causing compressive symptoms or cosmetic concerns; 5. Euthyroid hormonal profile before treatment; 6. Patient preference for a minimally invasive treatment or contraindications to surgery under general anesthesia.

Patients were excluded in cases of: 1. Nodules with suspicion of malignancy; 2. Bethesda III–VI at FNAB; 3. Nodules with significant retrosternal extension or in which an adequate safety margin could not be achieved despite hydrodissection; 4. Pregnancy or lactation; 5. Severe coagulation disorders or cardiopulmonary disease; 6. Pre-existing contralateral to the target site vocal cord paralysis.

Available treatment options, including surgery and thermal ablation, were discussed with eligible patients, and the final treatment decision was individualized according to clinical and nodule characteristics and patient preference.

#### 2.1.2. Pre-Ablation Assessment

Baseline data collection included demographic characteristics, medical history, presenting symptoms, and current medication use. Routine pre-procedural laboratory testing included complete blood count, coagulation profile, and thyroid function assessment (thyroid-stimulating hormone, free triiodothyronine and thyroxine, thyroid peroxidase antibodies, and thyroglobulin antibodies). Thyroid function tests were repeated at the 3-month follow-up.

All patients underwent cervical US evaluation and had at least one benign FNAB result prior to treatment. The US examination assessed nodule characteristics, such as the presence of solid and cystic components, internal vascularity, and well-defined margins. Additionally, US assessment included measuring the dimensions of the nodules and calculating their volume using the ellipsoid formula: V = πabc/6, equivalent to 0.524 × a × b × c, where V denotes volume and a, b, and c represent the maximum longitudinal, transverse, and anteroposterior diameters, respectively. Patients receiving anticoagulant therapy were advised to discontinue medication 2–7 days prior to ablation, depending on the pharmacokinetics of their medication.

Compressive symptoms severity was self-assessed by the patient using a 0–10 visual analogue score (VAS) scale, ranging from no neck symptoms (0) to the most severe neck symptoms (10). Cosmetic concerns were assessed by an independent physician using a 4-point cosmetic scoring system (1: no palpable nodule; 2: palpable but not visible nodule; 3: visible nodule during neck extension or swallowing; and 4: always visible nodule).

Patient’s pain level during and after the procedure was assessed using a 0–10 VAS scale, where 0 denotes no pain and 10 refers to the maximum conceivable pain. Potential side effects were also documented. Vocal cord mobility was evaluated in all patients using flexible laryngoscopy both prior to and following the procedure.

### 2.2. Statistics

Continuous variables were assessed for normality using the Shapiro–Wilk test and are presented as mean ± standard deviation (SD) or median (interquartile range/IQR), as appropriate, whereas ordinal variables are presented as median (IQR). Paired comparisons were performed using the Wilcoxon signed-rank test. A two-sided *p* value < 0.05 was considered statistically significant. All data were statistically analyzed using SPSS software for Windows version 27.0 (SPSS Inc., Chicago, IL, USA).

Volume-related outcomes were primarily analyzed at the nodule level, with each treated nodule paired with its corresponding baseline measurement for longitudinal comparisons. As five patients had two treated nodules, a sensitivity analysis was additionally performed using one nodule per patient; in patients with multiple treated nodules, the nodule with the larger baseline volume was selected. This analysis was performed to assess whether the inclusion of multiple nodules from individual patients materially influenced the volume-related outcomes.

### 2.3. Technical Implementation Protocol

All procedures were performed by an ENT–head and neck surgeon with fellowship training and extensive experience in thyroid surgery, as well as prior experience in head and neck ultrasonography. Before initiating independent thyroid MWA practice, the operator had undertaken dedicated training through hands-on MWA courses and observation of procedures performed by experienced operators at international centers.

#### 2.3.1. Equipment and Set-Up

Microwave ablation was performed using a 2450 MHz microwave generator (ECO Medical Technology Co., Ltd., Nanjing, China) equipped with a disposable, internally cooled with normal saline, 17-G antenna (10 cm length, 3 mm active tip). All procedures were performed under continuous real-time US guidance using a Philips CX50 ultrasound system (Philips Healthcare, Best, The Netherlands) equipped with an L12-3 high-frequency linear-array transducer.

**Figure 1:**
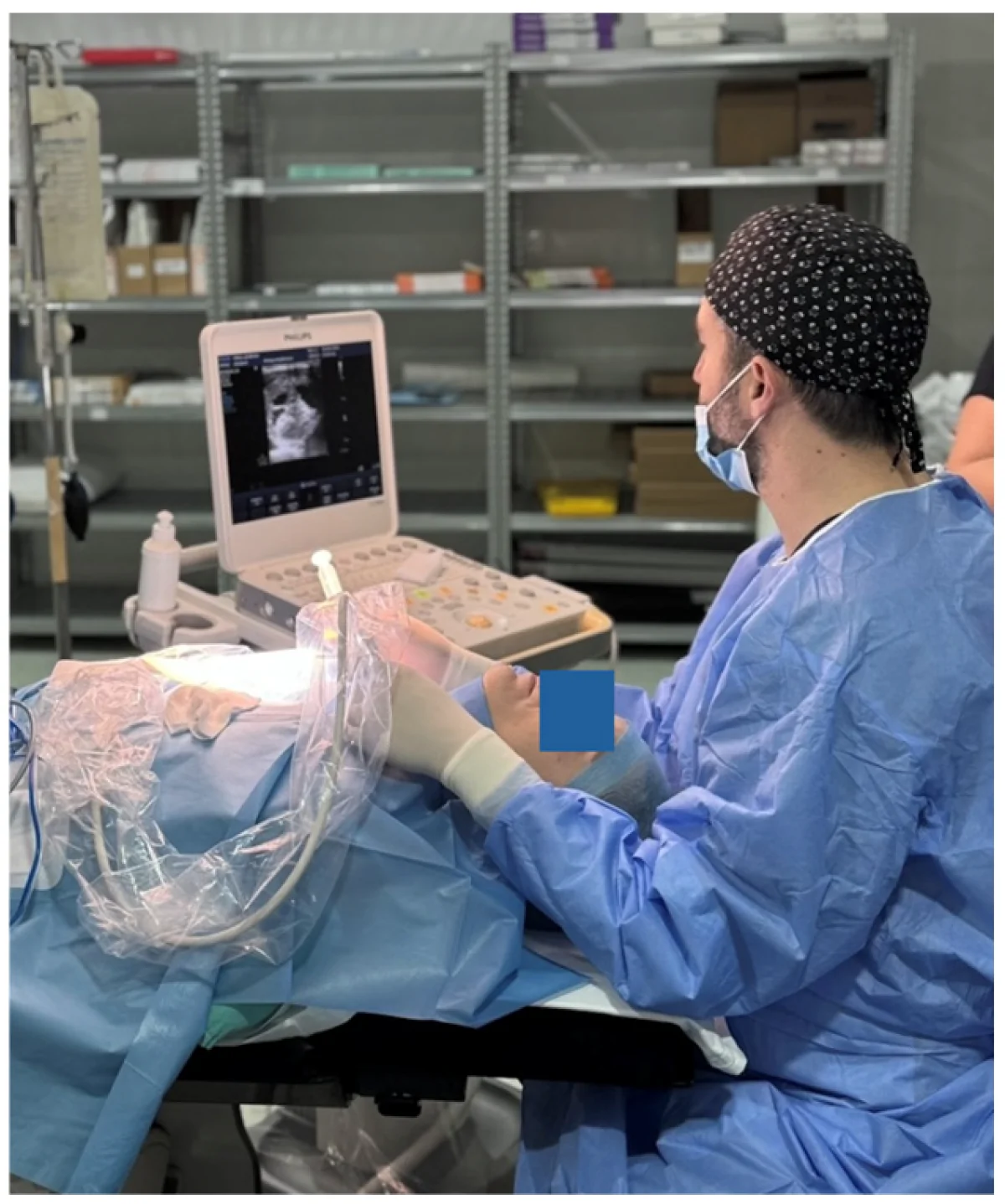
Operator positioning during ultrasound-guided microwave ablation, illustrating the relative positions of the operator, patient, and ultrasound system.

Patients were positioned in a supine position with mild neck extension throughout the procedure. Vital signs, including heart rate, blood pressure, and oxygen saturation were continuously monitored. The surgeon was positioned at the patient’s head allowing ergonomic bimanual manipulation of the instruments with equivalent access to both the right and left thyroid lobes (Figure 1).

#### 2.3.2. Anesthesia

The neck area was disinfected and surgical wipes were placed. All procedures were performed under local anesthesia. In selected anxious patients, a sublingual diazepam tablet (5 or 10 mg) was administered approximately 30 min prior to the procedure. Local anesthesia (lidocaine 2%) was initially injected subcutaneously into the puncture site extending to the area between the thyroid capsule and strap muscles, in order to protect adjacent structures from thermal damage and minimize pain or discomfort. Patients remained awake throughout the procedure, allowing immediate detection of voice changes or excessive pain.

#### 2.3.3. Hydrodissection

**Figure 2:**
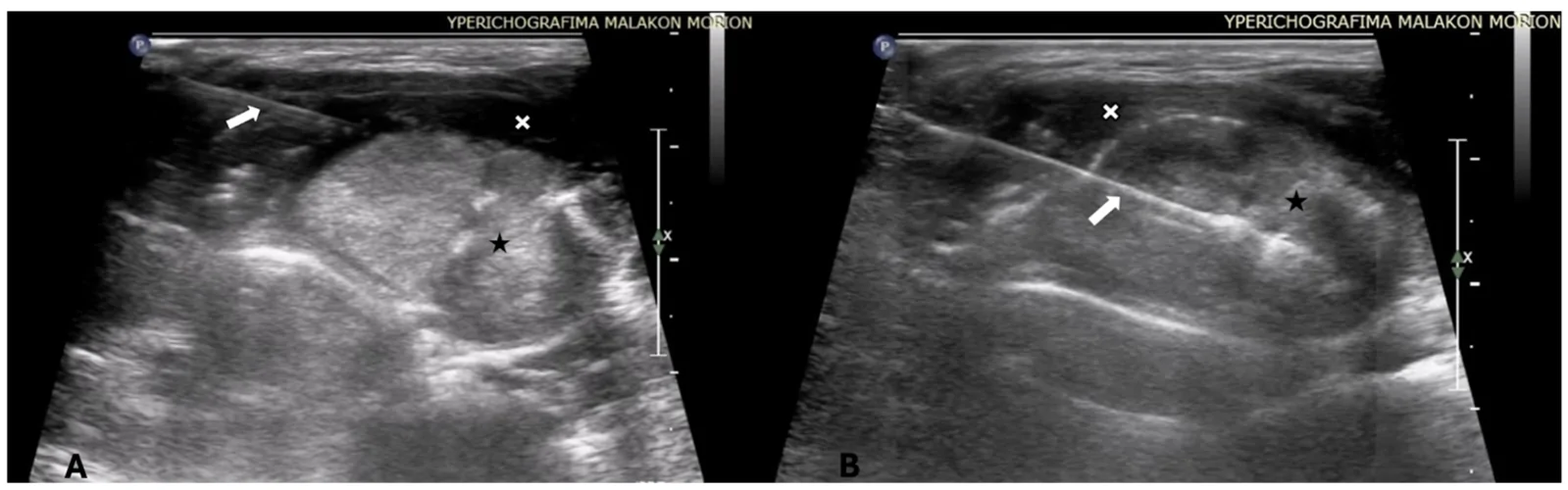
(**A**). Transverse ultrasound image demonstrating anterolateral hydrodissection prior to microwave ablation in a solid nodule of the right thyroid lobe. The needle was inserted through the thyroid isthmus, and fluid was injected to create a barrier separating the thyroid capsule from the adjacent structures. The thyroid nodule (asterisk), hydrodissection plane (x), and needle (arrow) are indicated. (**B**). Trans-isthmic approach for microwave ablation. A transverse ultrasound image demonstrating insertion of the antenna through the thyroid isthmus and ablation of a solid nodule in the right thyroid lobe. The thyroid nodule (asterisk), microwave antenna (arrow), and hydrodissection plane (x) are indicated.

Hydrodissection (HD) is an essential preliminary step prior to ablation to protect vital structures in proximity to the ablation area such as the recurrent laryngeal nerve, trachea, esophagus, strap muscles, and carotid sheath. Hence, HD using a cold 5% dextrose solution was systematically performed. Under US guidance, the fluid was slowly injected into the designated area to create a safe separation plane between the thyroid capsule and surrounding tissues. Based on the location of the targeted nodule, anterior, lateral, and/or posterior HD was performed (Figure 2A). The volume of injected fluid varied depending on nodule characteristics and patient’s anatomy. The aim was to inject sufficient volume to create a distance of at least 2–3 mm, which should be maintained throughout the ablation process. Repeated or continuous HD was performed during the procedure as necessary to maintain this liquid buffer.

#### 2.3.4. Antenna Insertion

Prior to the insertion of the microwave antenna, in predominantly cystic nodules, fluid aspiration was performed to improve treatment efficacy. In our practice, we perform initial skin puncture using a broad needle prior to the insertion of the antenna, which yields a better cosmetic outcome compared to a skin incision made with a scalpel. A trans-isthmic approach was preferentially used to improve antenna stability and visualization on US, as well as to minimize the risk of damage to vital surrounding structures. After ensuring that the tip of the antenna is located at the optimal area within the nodule, the microwave energy was delivered (Figure 2B). Microwave power was selected according to nodule size and proximity to critical structures. A power setting of 30 W was used for most nodules. Lower power (20 W) was preferred for smaller nodules and when ablating areas close to the thyroid capsule or critical adjacent structures, particularly the recurrent laryngeal nerve and trachea. Higher power (40 W) was reserved for larger nodules, particularly when treating their central portions away from critical structures. These settings served as a pragmatic framework and were dynamically adjusted during the procedure rather than applied according to rigid predefined size thresholds.

#### 2.3.5. Moving-Shot Technique

The moving-shot technique was routinely applied in our practice, particularly given the relatively large volume of many treated nodules. Under US guidance, the antenna was initially inserted into the deepest portion of the lesion, and the ablation began, while the antenna was gradually retracted slowly until reaching the nodule margin. During the procedure, a gradual hyperechogenic transformation of the targeted tissue was observed. Then, the antenna was superficially repositioned and pulled along the long axis of the nodule again. After multiple repeated movements in the same US section resulting in complete ablation of the tissue in this plane, the angle of the antenna was changed, and the procedure was repeated.

The antenna tip should be visualized during the whole ablation session. Any voice alteration prompted immediate cessation of energy delivery and reassessment of the procedure. If pain or discomfort occurred during the session, MWA was temporarily interrupted and repeated HD was performed before resuming. The ablation was considered complete when the entire thyroid nodule had turned into a hyperechoic area.

#### 2.3.6. Post-Procedural Assessment

At the end of the procedure, mild cold compression was applied to the neck for 20 min and patients were monitored in the hospital for the next few hours. Paracetamol 1 g was administered as needed, usually over a period of 1–3 days. Immediate return to normal oral intake was allowed. In our clinical practice, anti-inflammatory medications, steroids or antibiotics are not routinely prescribed. Routine post-ablation laryngoscopy was performed in all patients. Follow-up appointments were scheduled at 1, 3, 6, and 12 months thereafter. For the present early analysis, outcomes through the 3-month visit were included, while longer-term follow-up remains ongoing.

**Figure 3:**
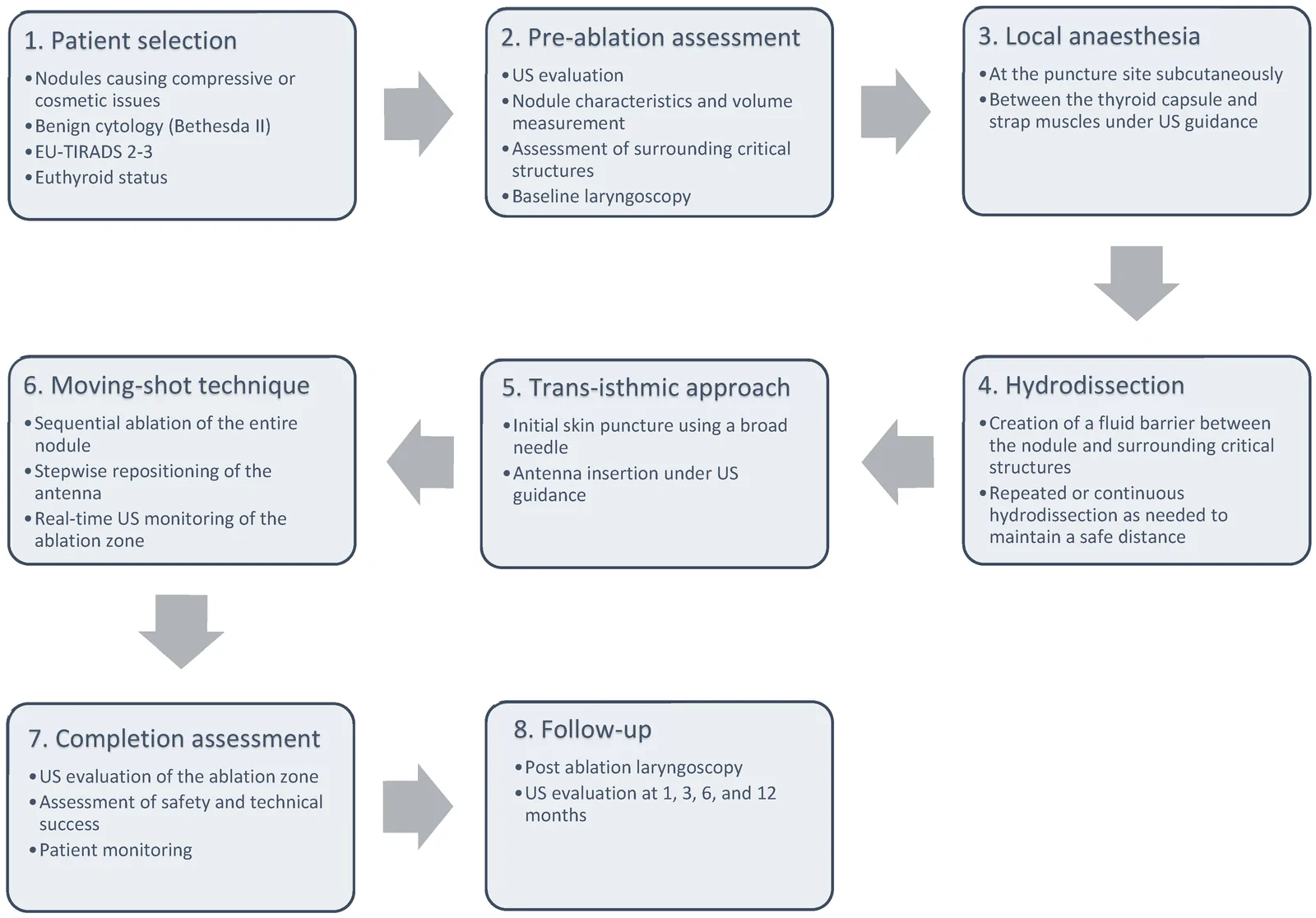
Workflow of ultrasound-guided microwave ablation for benign thyroid nodules.

Figure 3 illustrates the overall workflow for the structured implementation of US-guided MWA, from patient selection to post-procedural follow-up. The key practical recommendations derived from our implementation protocol and current evidence are summarized in Table 1.

## 3. Results

Twenty-five consecutive patients (5 males and 20 females) with a mean age of 53.2 ± 16.5 years (range, 28–86 years) were included in the analysis. A total of 30 benign thyroid nodules were treated with US-guided MWA. Their baseline characteristics are presented in Table 2.

### 3.1. Procedural Characteristics

Microwave ablation was successfully completed in all 25 patients with a mean ablation time of 4.8 min ± 3.3 (range, 1.1–15.4 min). All patients tolerated the procedure under local anesthesia well. In 9 out of 25 patients, a low-dose sublingual diazepam tablet was administered prior to the procedure, as a precautionary additional measure to control anxiety and ensure optimal tolerance. Throughout the procedure, patients remained awake and conscious, allowing for continuous communication to check for intra-procedural issues or complications such as hoarseness. All patients were discharged within 24 h in accordance with institutional protocol.

### 3.2. Safety

No major complications, including permanent recurrent laryngeal nerve injury, esophageal injury, or major bleeding, were observed. Likewise, there were no cases of temporary hoarseness, vagal reaction, dysphagia or skin burns. Two patients (8%) developed minor ecchymosis that resolved spontaneously within a few days. Mild localized pain occurred in 9 out of 25 patients and was managed conservatively.

Procedural pain was generally mild and decreased significantly by the following day (*p* = 0.001) (Table 3). No patient reported voice alteration after MWA. Flexible laryngoscopy performed one week after treatment confirmed normal vocal cord mobility in all patients.

### 3.3. Early Clinical Outcomes

Thirty benign thyroid nodules were treated and progressive reduction in nodule volume was observed during follow-up (Table 3). Median nodule volume decreased from 12.77 mL (IQR, 4.13–17.74) at baseline to 3.81 mL (IQR, 1.31–7.60) at 3 months after MWA, corresponding to a mean volume reduction ratio (VRR) of 63.8 ± 14.7% at 3 months. Significant improvements were also observed in compressive symptoms and cosmetic scores at 3 months (both *p* < 0.001). Thyroid function remained preserved in all patients, with no cases of new-onset hypothyroidism.

A sensitivity analysis performed using only the larger baseline nodule from each patient (*n* = 25) yielded comparable findings, with a median volume reduction from 14.11 mL (IQR, 9.97–18.58) at baseline to 5.34 mL (IQR, 2.83–8.20) at 3 months (*p* < 0.001) and a mean 3-month VRR of 63.2 ± 15.0%.

## 4. Discussion

Although MWA has been increasingly adopted worldwide, there is limited literature focusing on its practical implementation within ENT departments. Most published series primarily report clinical outcomes, whereas detailed descriptions of procedural standardization, safety strategies, and implementation are scarce. The present study addresses this gap by describing the early implementation of ultrasound-guided MWA for benign thyroid nodules in a tertiary ENT center. Our early experience supports the feasibility and short-term procedural safety of ultrasound-guided MWA for benign thyroid nodules in carefully selected patients, with favorable early clinical outcomes and excellent tolerability under local anesthesia.

Careful patient selection is paramount for effective thyroid ablation. Although most guidelines primarily refer to the use of RFA, similar indications also apply to MWA [12,13,14]. Importantly, although the indications for thyroid thermal ablation have expanded to selected malignant lesions, novice operators should initially restrict their practice to carefully selected benign nodules with favorable anatomical characteristics [14,15]. Currently, there are no definitive nodule size criteria established, as the presentation of symptoms may differ based on the nodule’s location and patient’s characteristics. Of note, large nodules with a volume greater than 20–30 mL may need multiple ablative sessions [16]. The final decision for treatment should be made after discussion with the patient about benefits, complications and alternative treatment options such as surgery.

In addition to proper patient selection, meticulous procedural technique is critical for safe implementation. Unlike conventional thyroid surgery, thermal ablation can be performed without the need for general anesthesia, allowing the procedure to be undertaken outside the operating theatre. However, it is essential for the procedure to be done in an appropriately equipped setting with continuous monitoring of the patient’s vital signs and immediate access to surgical and anesthetic support should an emergency arise. In our experience, local anesthesia is adequate for most patients and allowed them to remain awake and cooperative throughout the procedure. This facilitates the immediate recognition of pain or voice changes that may indicate thermal irritation, thereby allowing prompt interruption of energy delivery and reassessment of the ablation field. Nevertheless, mild conscious sedation may occasionally be required in selected cases, as recommended by the European Thyroid Association guidelines [12]. Alternatively, we found that pre-ablation sublingual administration of diazepam tablet was particularly beneficial in selected anxious patients, as it improved procedural tolerability without compromising patient cooperation and communication throughout the procedure.

Attention to several technical considerations is fundamental to the safe implementation of thyroid MWA. In our unit, a trans-isthmic approach is preferentially used because it provides good antenna visualization and stability and minimizes the risk of thermal injury to vital structures [17]. Alternatively, in cases where this access is not feasible, a lateral access can be chosen. Furthermore, considering the relatively large volume of many treated nodules, the moving-shot technique was preferentially used instead of a single-point technique. This approach allows sequential ablation of multiple nodular units, while protecting surrounding structures of the neck [14,17].

To further mitigate the risk of thermal injury, HD is one of the most important safety maneuvers during thyroid MWA. Rather than a single preparatory step, it should be regarded as a dynamic process, because the injected fluid is gradually absorbed during ablation, requiring repeated HD, whenever the protective fluid plane becomes inadequate. In our experience, repeated HD was also often required whenever patients reported transient discomfort, despite apparently adequate fluid margin on US. Continuous HD has recently been proposed as an alternative technique for maintaining a stable fluid barrier throughout prolonged procedures [18].

Microwave ablation has consistently exhibited a favorable safety profile, with the majority of procedure-related adverse events being minor, transient, and self-limiting. In a large retrospective series by Liang et al. [19], encompassing 4494 procedures, major complications were infrequent; hemorrhage was observed in 1.0% of cases, transient hoarseness in 0.4%, and symptomatic aseptic necrosis in 0.16%, with no reported cases of permanent recurrent laryngeal nerve injury. Similarly, a meta-analysis by Zheng et al. [20] reported a pooled major complication rate of 4.8%, identifying transient voice alterations as the most common serious event. Our clinical observations align with this evidence, as our cohort experienced only minor, self-limiting complications, with no occurrences of major adverse events; however, the small sample size of the present study precludes conclusions regarding uncommon or rare adverse events.

Although RFA remains the most extensively studied thermal ablation modality and is recommended as the first-line minimally invasive treatment option for selected benign thyroid nodules [12], previous studies have demonstrated comparable efficacy and safety profiles between RFA and MWA [14,21,22]. Our choice of MWA was, therefore, not based on the assumption of superiority over RFA, but rather on its technical characteristics, including the ability to generate larger ablation zones within shorter procedural times and its suitability for the patient population encountered in our practice.

Nowadays, MWA is performed by doctors of different specialties and most published data come from interventional radiologists, endocrinologists and endocrine surgeons. ENT surgeons may also be well positioned to integrate thyroid thermal ablation into multidisciplinary thyroid practice owing to their familiarity with cervical anatomy, recurrent laryngeal nerve preservation, and laryngeal function assessment. Although US-guided thermal ablation has a low incidence of major complications, clinically significant events such as hemorrhage, recurrent laryngeal nerve dysfunction, or, in rare cases, airway compromise may occur [19]. Within this framework, an ENT surgeon can provide a comprehensive peri-procedural approach, including routine laryngoscopy to assess vocal fold mobility, and immediate surgical management in cases of complications such as hematoma or airway compromise. Furthermore, close collaboration between the ENT, radiology, and endocrinology departments has been fundamental to our implementation strategy, ensuring continuity of care and supporting the multidisciplinary management of patients with thyroid nodules.

As thyroid thermal ablation becomes increasingly adopted, standardization of operator training is essential. Current guidance underlines the value of appropriate experience in thyroid procedures and ultrasonography, training in thermal ablation techniques, and ability to prevent and manage complications [11,13]. Although the present study was not designed to formally assess the learning curve, our experience supports the importance of such structured preparation before independent implementation of MWA in clinical practice.

Several limitations of this study should be acknowledged. First, the relatively small sample size and single-center, single-operator design limit the generalizability of our findings. Importantly, the study was not powered to detect uncommon or rare adverse events; therefore, the absence of major complications in this cohort should be interpreted cautiously and cannot establish the overall safety profile of MWA. Second, the absence of a control or comparison group precludes conclusions regarding the comparative effectiveness of MWA relative to surgery, RFA, or other treatment modalities. Third, the short follow-up duration limits assessment of sustained volume reduction, nodule regrowth, and the potential need for repeat ablation. Although reduction in nodule volume provided an objective imaging-based efficacy outcome, improvement in compressive symptoms was assessed using a patient-reported symptom score, while cosmetic improvement was based on physician assessment. Therefore, these clinical endpoints were not complemented by objective functional measures. The present findings should therefore be interpreted as early clinical outcomes primarily supporting the feasibility and short-term procedural safety of MWA implementation rather than as evidence of long-term efficacy or comparative effectiveness.

## 5. Conclusions

Our early experience supports the feasibility and short-term procedural safety of ultrasound-guided MWA for selected patients with benign thyroid nodules. The technique can be integrated within an ENT department using standardized procedural and safety protocols. Successful implementation depends not only on the technology itself but also on careful patient selection, meticulous technique, and structured operator training. Beyond its favorable early clinical outcomes, this study provides a practical framework for centers seeking to establish thyroid MWA programs.

## Figures and Tables

**Table 1:** Key practical recommendations for safe implementation of thyroid MWA based on current evidence and our institutional experience.

Pearl	Practical Recommendations
Patient selection	Compliance with current guidelines for patient selection reduces the risk of ineffective MWA. Novice operators should initially perform MWA in carefully selected benign nodules with favorable size and location.
Anesthesia	The procedure is well tolerated under local anesthesia in most patients. Consider administering a low-dose sublingual diazepam tablet prior to MWA in selected anxious patients.
Hydrodissection	Always maintain an adequate fluid buffer. In our practice, repeat HD is performed whenever the protective fluid plane becomes thinner than 2–3 mm or the patient reports pain or discomfort.
Antenna	Never advance the antenna or start ablation unless the antenna tip is completely visible.
MWA technique	For large nodules, prefer the moving-shot technique with multiple overlapping ablation zones, rather than increasing the power output. Adjust power according to nodule size and proximity to critical structures, using lower power near vulnerable structures.
Communication	Maintain continuous verbal communication throughout the procedure, as patient-reported voice alteration or discomfort is usually the first sign of RLN irritation or thermal injury to adjacent structures. Stop MWA immediately, if voice changes occur, and reassess the ablation field.
Laryngoscopy	In our practice, routine pre- and post-ablation laryngoscopy is performed in all patients for the early identification of vocal cord dysfunction. Normal voice does not exclude impaired vocal cord mobility.

MWA: microwave ablation; HD: hydrodissection; RLN: recurrent laryngeal nerve.

**Table 2:** Baseline characteristics of patients and treated thyroid nodules.

Variable	Value
Number of patients	25
Age (years)	53.2 ± 16.5
Sex, *n* (%)	
Female	20 (80%)
Male	5 (20%)
Number of nodules	30
Nodule position, *n* (%)	
Right	16 (53.3%)
Left	11 (36.7%)
Isthmus	3 (10%)
Nodule composition, *n* (%)	
Predominantly solid	18 (60%)
Predominantly cystic	3 (10%)
Mixed	9 (30%)
Nodule volume (mL)	12.77 (4.13–17.74)
<10 mL	11 (36.7%)
10–20 mL	14 (46.7%)
>20–30 mL	2 (6.7%)
>30 mL	3 (10%)
Compressive symptom score	4.3 ± 2.1
Cosmetic score	3 (2–4)

Data are presented as mean ± SD, median (IQR), or *n* (%), as appropriate. *n*: number; SD: standard deviation; IQR: interquartile range.

**Table 3:** Early clinical efficacy and safety outcomes following ultrasound-guided MWA.

Outcome	Baseline/Procedural	Follow-Up	*p* Value
Nodule volume, mL	12.77 (4.13–17.74)	9.65 (2.63–13.53), 1 month	<0.001
		3.81 (1.31–7.60), 3 months	<0.001
VRR, %	—	30.7 ± 16.9, 1 month	—
		63.8 ± 14.7, 3 months	—
Compressive symptom score	4.3 ± 2.1	2.0 ± 1.5, 3 months	<0.001
Cosmetic score	3 (2–4)	2 (2–3), 3 months	<0.001
Pain score	1.7 ± 1.7	0.7 ± 1.3, day 1	0.001
New-onset hypothyroidism, *n* (%)	—	0 (0%)	—
Major complications	—	0 (0%)	—
Ecchymosis	—	2 (8%)	—

Data are presented as mean ± SD, median (IQR), or *n* (%), as appropriate. Volume-related outcomes were analyzed at the nodule level (*n* = 30), whereas clinical, functional, and safety outcomes were analyzed at the patient level (*n* = 25). MWA: microwave ablation; VRR: volume reduction ratio; SD: standard deviation; IQR: interquartile range; *n*: number.

## Data Availability

Data are available upon reasonable request. The data are not publicly available due to privacy considerations related to the small clinical cohort.
